# The impact of enhanced recovery after surgery on inflammatory indicators and prognosis related to complex appendicitis in children

**DOI:** 10.3389/fped.2023.1261191

**Published:** 2023-10-20

**Authors:** Jian-guo Zhang, Hao-wei Li, Xiang-ming Wu, Hai-bin Yu, Yan-hui Liu, Lin Qi, Yu Bai, Lin Yang, Hai-long Zhang, Xiao-yun Wang, Yue-qi Jia

**Affiliations:** ^1^ Pediatric Surgery, Inner Mongolia Maternal and Child Health Care Hospital, Hohhot, China; ^2^ Pediatric Surgery, The Fourth Hospital of Baotou, Baotou, China

**Keywords:** ERAS, laparoscopic surgery, inflammatory indicators, complex appendicitis, children

## Abstract

**Objective:**

To explore the application effect of enhanced recovery after surgery (ERAS) perioperative plan in the treatment of complex appendicitis in children, and further enrich the implementation plan of ERAS in the field of pediatric surgery.

**Method:**

This study selected 122 children who underwent laparoscopic complex appendectomy at Inner Mongolia Maternal and Child Health Hospital and Baotou Fourth Hospital from August 2018 to July 2022, and randomly divided them into a traditional surgery group (TS) and an enhanced recovery surgery group (ERAS). The changes of white blood cell (WBC), hypersensitive C-reactive protein (CRP), pro Calcitonin (PCT) and interleukin 6 (IL-6) before and after surgery were compared. The degree of pain, recovery time of intestinal function, length of hospital stay, hospital costs, postoperative complications and parental satisfaction were compared between the two groups.

**Result:**

The WBC and CRP levels in the ERAS group at 6 h after surgery, as well as the IL-6 levels on the 3rd day after surgery, were lower than those in the TS group. Meanwhile, the analgesic effect of ERAS group at 3 h and 6 h after surgery was better than that of TS group. And the ERAS group had a shorter postoperative first exhaust time, fewer overall hospital stays, and lower hospitalization costs. In addition, the ERAS group had high parental satisfaction during hospitalization. There was no statistically significant difference in postoperative complications between the two groups of children.

**Conclusion:**

ERAS can promote postoperative recovery of children, reduce surgical stress, save family medical expenses, alleviate the pain of children, and improve parental satisfaction. It is a safe and effective method for treating complex appendicitis in children.

## Introduction

The concept of enhanced recovery after surgery (ERAS) was proposed by Dr. Henrik Kehlet in 1990 to improve the patient's perioperative plan (1). The purpose is to promote postoperative patient recovery through various perioperative interventions, thereby reducing complications and overall medical costs. Through continuous research and improvement in recent years, these plans have played a positive role in almost all aspects, from simple daily surgeries to complex surgeries, becoming effective tools for surgeons to accelerate patient recovery and improve prognosis.

The children's appendix wall is thin, the ileocecal region is rich in lymphoid tissue, the greater omentum is poorly developed, and the peritoneal cavity area is relatively large, so it is prone to suppuration and perforation, thus developing into complex appendicitis (CAA). At the same time, peritonitis, sepsis, intestinal obstruction, abscess formation, and fertility problems are combined (2), which leads to longer hospital stays and additional costs. Abdominal inflammation in children is difficult to control and limited, with severe systemic poisoning symptoms, a high incidence of complications, and a significantly higher mortality rate than in adults. Scientific perioperative preparation can help the smooth progress of the surgery and accelerate the recovery of the patient. Therefore, the concept of enhanced recovery after surgery has certain research significance in the treatment of complex appendicitis in children and improving their prognosis.

## Materials and methods

### General information

This study selected 122 children who underwent laparoscopic complex appendectomy at Inner Mongolia Maternal and Child Health Hospital and Baotou Fourth Hospital from August 2018 to July 2022. The cases were randomly divided into the traditional surgery group (TS) and the enhanced recovery after surgery (ERAS) group according to the random number table. Briefly, all cases are numbered first, then three digits are taken as corresponding numbers according to the random number table, and finally, the random number is sorted from smallest to largest, so as to achieve complete random grouping. 62 children who underwent perioperative intervention according to the accelerated rehabilitation surgical standards were used as the experimental group (ERAS group), while 60 children who underwent traditional perioperative intervention were used as the control group (TS group). The preoperative general information (including gender, age, appendix case classification—purulent and perforated) of all patients was collected. This study was approved by the Ethics Committee of the Maternal and Child Health Hospital of Inner Mongolia Autonomous Region (Approval No. [2020] Lun Han Shen No. [074–2]) and obtained informed consent from patients' guardians.

### Diagnostic and exclusion criteria for inclusion in this study

***Inclusion criteria:*** (1) Having signs of appendicitis (symptoms such as fever, abdominal pain, vomiting, etc.); (2) Preoperative blood and imaging examinations support the diagnosis of appendicitis; (3) The pathological type of appendicitis is based on surgical and pathological diagnosis (purulent, gangrenous, perforated); (4) The surgical procedure is laparoscopic appendectomy.

***Exclusion criteria:*** (1) Patients over 15 years old; (2) Previous history of abdominal surgery; (3) Having related symptoms for more than 2 weeks or a history of chronic appendicitis; (4) Merge with other diseases or have anatomical abnormalities; (5) Patients who are unable to tolerate minimally invasive surgery or who fail to undergo minimally invasive surgery and switch to open surgery.

### Surgical methods and perioperative management

#### Surgical methods

Both groups of children underwent laparoscopic surgery. After completing routine preoperative preparation, an incision was made along the upper edge of the child's navel, and a CO_2_ pneumoperitoneum (pressure 8–12 mmHg) was established using a pneumoperitoneum needle. A 5 mm or 10 mm Trocar was implanted according to the child's age and development, and the abdominal cavity was routinely explored using laparoscopy. Under laparoscopic direct vision surgery, a trocar was used to puncture the operating hole along the midline of the clavicle, approximately 2 cm below the right costal margin, and one-third of the “reverse Mack's point” on the line connecting the left lower abdominal navel and the anterior superior iliac spine. After exploring the situation in the abdominal cavity again, adjusted the operating table, changed the child's head to a high and low lying position on the left side (leaning 15–30° to the left), carefully separated the adherent appendix and surrounding tissues, separate and ligate the mesentery of the appendix, dissociate it to the root of the appendix about 0.5 cm away from the ileocecal part and clamp it with an absorbable clamp, cut off the appendix, and treat the appendix stump. Use a suction device and clean gauze to clean the pus in the abdominal cavity. After removing the appendix, recheck the residual end of the appendix and the condition of the abdominal cavity. If necessary, rinse the abdominal cavity, and send the appendix specimen to the pathology department.

#### Comparison of perioperative management methods between two groups of pediatric patients

The preoperative, intraoperative, and postoperative management plans of the ERAS group are different from those of the TS group. Through the study of the ERAS consensus and guidelines (3) and the retrieval of relevant research literature, this study developed an ERAS protocol suitable for this study, and compared it with the TS group protocol (Table 1).

**Table 1:** Differences in perioperative measures between TS group and ERAS group.

	TS group	ERAS group
Propaganda and education	Regular admission education	Popularize disease related knowledge, introduce ERAS concept, introduce specific procedures and precautions for admission, examination, surgery, and discharge
Preoperative	① Strictly fasting for 12 h before surgery and drinking water for 6 h before surgery, or strictly fasting and drinking water at the time of self-diagnosis. ② Perform intestinal preparation such as enema, and place a nasogastric tube if necessary. ③ Routine preoperative visits are usually based on parents providing information to check the condition of the child. ④ After entering the operating room, anesthesia will be administered.	① Fasting for 6 h before surgery, and drinking water for 2 h before surgery. Prior to this, give the child 12.5% glucose water 5 ml/kg for consumption (up to 100 ml). ② Try to minimize or avoid preoperative intestinal preparation, and do not leave a nasogastric tube. ③ Anesthesiologists patiently visit, introduce themselves, inquire about the patient's name, age, etc., and have good interaction with the patient. ④ Induce anesthesia before entering the operating room.
Intraoperative	① No special heating device, ordinary sterile sheet. ② Anesthesia has no special emphasis and is mainly evaluated by the anesthesiologist during surgery. ③ Retain drainage tube and catheter according to the situation. ④ Regular use of absorbable suture for subcutaneous suture.	① During surgery, devices such as insulation blankets and hot air fans should be used to maintain the child's body temperature at least 36 °C and prevent excessive body temperature. ② The chief surgeon communicates fully with the anesthesiologist, adjusts the anesthetic medication in a timely manner, and fully shortens the postoperative recovery time. ③ Do not place any drainage and do not place a catheter. ④ Inject ropivacaine (0.75%; 10 ml) into each incision before subcutaneous suturing with absorbable suture, and dilute and inject 1–2 ml according to the size of the incision.
Postoperative	① Provide painkillers as needed (regardless of type). ② There are no special requirements for postoperative fluid replacement, and the required amount of fluid should be replenished. ③ Start moving out of bed on the first day after surgery. ④ After exhausting, one can drink water and consume a small amount of liquid food.	① Analgesic effects of acetaminophen suppositories, avoiding the use of morphine like drugs. ② Strictly follow the principle of fluid replacement, try to reduce the amount of intravenous crystal fluid supplementation for children, and group fluid replacement according to the status of the children. ③ Bed activity can begin 6 h after surgery, and on the first day after surgery, get out of bed and develop an activity plan. ④ After the child wakes up 6 h under anesthesia, a small amount of water can begin to flow, gradually increasing after no discomfort.

The specific scheme of preoperative anesthesia induction, intraoperative analgesia and fluid management were as follows: (1) Induced anesthesia: a. Sufentanil, 0.2–0.4 *μ*g/kg; b. Propofol, 2.5–4 mg/kg (1 month-3 years old), about 2.5 mg/kg (>8 years old); c. Vecuronium bromide,0.1–0.2 mg/kg and 0.02 mg/kg (intermittent administration was maintained during the operation); d. Remifentanil, 0.5–1 *μ*g/kg/min and 0.05–2 *μ*g/kg/min (intraoperative anesthesia maintenance); e. Dexmedetomidine, 0.2–0.7 *μ*g/kg/h. (2) Intraoperative analgesia (including regional/local analgesia) ropivacaine (0.75%; 10 ml) was injected into incisions one by one before subcutaneous suture with absorbable line. The ropivacaine was diluted 1:1 with saline and injected into incisions according to the incision size. (3) Fluid management: Children with complex appendicitis are usually complicated by dehydration, electrolyte disturbance, hypoglycemia and other conditions after admission. Perioperative fluid rehydration should be calculated according to the specific situation of each child, and the urine volume of the child should be monitored and dynamically adjusted. During the operation, the fluid volume was calculated mainly according to the physiological requirements of the child, the amount of fasting water lost before the operation, the amount of loss during the operation, the amount of third space loss, and the amount of blood loss. The balance of isotonic electrolyte solution was the main method, and the adjustment of electrolyte and blood glucose level was referred to before the operation.

### Postoperative treatment

Both groups of children were treated with antibiotics of the same grade after surgery. The temperature of the children was observed to drop to normal for 2 days, and the inflammatory indicators were tested to normal before discontinuing antibiotics. After discharge, the patients were followed up for 3 months. The main follow-up methods are outpatient follow-up, ultrasound examination of the appendix stump and intra-abdominal conditions.

### Observation indicators

Record the first postoperative exhaust time, hospitalization days, hospitalization expenses, postoperative complications, and parental satisfaction of the two groups of children.

The pain degree (VAS scoring method) at 3 h, 6 h and 12 h after surgery was recorded (4). The pain level was evaluated using the VAS scoring method, with no wound pain (0–2 points), mild pain (3–5 points), significant but tolerable pain (6–8 points), and unbearable pain (8–10 points). At the first evaluation, the doctor and the parents of the child jointly describe and explain to the child.

The changes of white blood cell (WBC), hypersensitive C-reactive protein (CRP), interleukin 6 (IL-6), pro Calcitonin (PCT) and other related inflammatory indicators before, 6 h after surgery, and 3 days after surgery were recorded.

### Discharge standard

The patient has no fever or normal blood count within 2 days, no discomfort after a normal or semi-liquid diet, no other complications, and the incision has recovered well.

### Satisfaction survey

When the child is discharged, the satisfaction level of the parents was understood and recorded. The evaluation content includes: (1) understanding of the child's disease; (2) Satisfaction with admission notification and communication; (3) Understanding of treatment plans; (4) The comfort level and crying situation of the patient during hospitalization; (5) The feelings and anxiety of family members during bedtime; (6) Overall satisfaction with the ward environment; (7) Satisfaction with the recovery level of the child; (8) Satisfaction with the attitude of medical staff; (9) Satisfaction with health education and discharge guidance; (10) Willing to recommend undergraduate programs to relatives and friends in need. The ERAS group was scored anonymously by the patient's family members, with satisfaction (7–9 points), relatively satisfied (4–6 points), and dissatisfied (1–3 points).

### Statistical methods

All statistical data were imported into SPSS 20.0 software for analyzing differences. The count data were represented by *n* (%) and a chi-square test was used to analyze the difference. The measurement data were represented by mean ± standard deviation (SD) and analyzed by *T*-test (conforming to normal distribution) and nonparametric tests (not conforming to normal distribution), respectively. *P *< 0.05 indicates a statistically significant difference.

## Results

### Baseline information

In total, 122 cases were included and divided into ERAS (*n* = 62) and TS group (*n* = 60). All of which successfully completed laparoscopic appendectomy without any conversion to open surgery. Baseline characteristics of patients in the two groups were listed in Table 2. The results showed that there were no significant differences in gender, age and pathological classification between the two groups (*P *> 0.05), which could be followed by comparison.

**Table 2:** Comparison of baseline characteristics in patients between ERAS group and TS group.

Characteristic	ERAS (*n* = 62)	TS (*n* = 60)	t/*χ*^2^	*P* Value
Gender, *n* (%)			0.265	0.607
Male	40 (52.63%)	36 (47.37%)		
Female	22 (47.83%)	24 (52.17%)		
Age, Month	88.645 ± 36.948	86.683 ± 38.885	0.286	0.776
Pathological classification, *n* (%)			0.032	0.859
Suppurative	32 (51.61%)	30 (50%)		
Perforation	30 (48.39%)	30 (50%)		

Data were expressed as mean ± standard deviation or *n* (%).

### The effect of ERAS perioperative regimen on postoperative inflammatory-related indicators in patients after surgery

To determine the effect of the ERAS perioperative regimen on postoperative inflammatory status in patients with appendicitis, we measured WBC, CRP, PCT, and IL-6 levels in patients with appendicitis before surgery, 6 h after surgery, and on the third day after surgery. The results showed that there were no significant differences in the levels of WBC, CRP, PCT and IL-6 between the TS and ERAS groups before operation (*P* > 0.05). Compared with the TS group, the patients in the ERAS group had lower WBC (*P *= 0.036) and CRP (*P *= 0.027) levels at 6 h after surgery, and lower IL-6 levels (*P *= 0.031) on the 3rd day after surgery, with a faster recovery rate (Table 3).

**Table 3:** Comparison of inflammatory indexes between the two groups.

Inflammatory indexes		ERAS (*n* = 62)	TS (n = 60)	t	*P* Value
WBC (×10^9^/L)	pre-operation	14.624 ± 4.8489	15.730 ± 5.6281	−1.164	0.247
	6 h after surgery	11.304 ± 4.100	13.131 ± 5.348	−2.121	0.036
	3rd day after surgery	7.936 ± 2.845	8.836 ± 4.862	−1.253	0.213
CRP (mg/L)	pre-operation	52.564 ± 50.929	61.847 ± 69.650	−0.842	0.401
	6 h after surgery	50.356 ± 42.650	71.823 ± 61.790	−2.239	0.027
	3rd day after surgery	15.977 ± 26.329	17.222 ± 26.302	−0.261	0.794
PCT (ng/ml)	pre-operation	6.369 ± 9.429	4.313 ± 10.596	1.133	0.259
	6 h after surgery	7.767 ± 8.620	6.431 ± 9.956	0.794	0.429
	3rd day after surgery	0.739 ± 0.971	0.816 ± 1.618	−0.319	0.750
IL-6 (ng/L)	pre-operation	121.882 ± 275.814	104.357 ± 223.727	0.385	0.701
	6 h after surgery	40.448 ± 69.362	52.902 ± 78.147	−0.932	0.353
	3rd day after surgery	6.051 ± 6.147	9.717 ± 11.623	−2.188	0.031

Values were expressed as mean ± standard deviation. WBC, white blood cell; CRP, C-reactive protein; PCT, pro Calcitonin; IL-6, interleukin 6.

### The impact of ERAS perioperative regimen on the pain of patients with appendicitis after surgery

Subsequently, we assessed postoperative pain in patients with appendicitis using the VAS scale (Table 4). Compared with the TS group, pain scores of appendicitis patients in the ERAS group were significantly lower at 3 h (3.807 ± 1.226 *vs*. 4.417 ± 1.279, *P *= 0.008) and 6 h (3.097 ± 1.327 *vs*. 3.6333 ± 1.365, *P *= 0.030) after surgery. The pain index of appendicitis patients in the ERAS group at 12 h after surgery was lower than that in the TS group, but there was no significant difference (*P *> 0.05). These results showed that the analgesic effect of the ERAS group at 3 h and 6 h after surgery was better than that of the traditional surgical group.

**Table 4:** Comparison of postoperative analgesia, postoperative intestinal exhaust time, postoperative hospital stay, overall hospitalization expenses, the incidence of postoperative complications and satisfaction of parents between the two groups.

	ERAS (*n* = 62)	TS (*n* = 60)	t/ χ²/z	*P* Value
Postoperative analgesia				
3 h after surgery	3.807 ± 1.226	4.417 ± 1.279	−2.690	0.008
6 h after surgery	3.097 ± 1.327	3.6333 ± 1.365	−2.201	0.030
12 h after surgery	3.807 ± 0.902	3.933 ± 0.899	−0.778	0.438
Intestinal exhaust time (hours)	17.581 ± 4.668	20.883 ± 8.501	−2.671	0.009
Postoperative hospitalization stay (days)	9.597 ± 3.375	10.967 ± 3.760	−2.119	0.036
Hospitalization expenses (Yuan)	11,722.602 ± 2,588.964	12,896.126 ± 3,426.475	−2.139	0.034
Postoperative complications, *n* (%)			0.119	0.730
Incidence	4 (6.45%)	3 (5.00%)		
Non-incidence	58 (93.55%)	57 (95.00%)		
Satisfaction, *n* (%)			−2.071	0.038
Satisfaction	48 (77.42%)	36 (60.00%)		
Relatively satisfaction	13 (20.97%)	22 (36.67%)		
Dissatisfy	1 (1.61%)	2 (3.33%)		** **

Data were expressed as mean ± standard deviation or *n* (%).

### The effect of ERAS perioperative regimen on postoperative intestinal exhaust, length of hospital stay, and hospitalization costs in patients after surgery

In order to further clarify whether there were differences in hospital stay and cost between the two groups of patients with appendicitis, we counted the first postoperative exhaust time of the patient and evaluated the hospitalization days and expenses according to the discharge standards. The results showed that the mean first exhaust time (17.581 ± 4.668 *vs*. 20.883 ± 8.501, *P *= 0.009) and length of hospital stay (9.597 ± 3.375 *vs*. 10.967 ± 3.760, *P *= 0.036) in the ERAS group were significantly shorter than those in the TS group. The corresponding hospitalization cost (11,722.602 ± 2,588.964 *vs*. 12,896.126 ± 3,426.475, *P *= 0.034) was also significantly reduced (Table 4). The patients in the ERAS group had a shorter postoperative first exhaust time, fewer overall hospital stays, and lower hospitalization costs, effectively accelerating the postoperative recovery level of patients, increasing patient comfort, and reducing economic pressure and parental burden.

### The effects of ERAS perioperative regimen on postoperative complications in patients

We recorded the occurrence of postoperative complications in two groups of appendicitis patients. There were 4 postoperative complications in the ERAS group, including 1 case of incision bleeding caused by children's postoperative activities;1 case of incision infection; 2 cases of abdominal pain after a small amount of liquid diet, and a small amount of gas-liquid level found on abdominal plain film, indicating incomplete intestinal obstruction. In the TS group, 3 postoperative complications occurred, with 1 case experiencing abdominal pain 5 days after surgery, and a small amount of fluid accumulation in the abdominal cavity was assisted in the examination; One case had abdominal pain 7 days after surgery, with ultrasound indicating abdominal abscess and elevated infection indicators. One case developed abdominal pain after a small amount of liquid diet, and a small amount of gas-liquid level was found on abdominal plain film, indicating incomplete intestinal obstruction. There was no significant difference in postoperative complications between the two groups of children (6.45% *vs*. 5%, *P *= 0.730) (Table 4).

### The effects of ERAS perioperative regimen on parental satisfaction

The ERAS group was scored anonymously by the patient's family members, with satisfaction (7–9 points), relatively satisfied (4–6 points), and dissatisfied (1–3 points). The satisfaction level of parents in the ERAS group was higher than that in the TS group (*P *= 0.038) (Table 4).

## Discussion

The ERAS concept refers to enhancing patient recovery and reducing the incidence of complications by optimizing perioperative plans (5). At present, there is an average of 23.8 protocols applied to adult surgery (6), and good results have been achieved. However, the development of standard perioperative protocols for enhanced recovery surgery in pediatric surgery is still relatively slow. Currently, research has confirmed that the ERAS concept can be safely applied to children undergoing routine surgery (7–10), but we still need a lot of research to explore and enrich truly applicable accelerated protocols for pediatric surgery.

The physical signs of appendicitis in children are numerous and not obvious. Inflammation in the abdominal cavity is prone to spreading, leading to complications such as perforation, peritonitis, and peripheral abscesses. In severe cases, sepsis may occur and endanger life (11). The research results compared the changes in four inflammatory indicators between the two groups of children before surgery, 6 h after surgery, and 3 days after surgery. There were differences in WBC at 6 h after surgery, CRP at 6 h after surgery, and IL-6 indicators on 3 days after surgery. Summary of the reasons: (1) Within 6 h after surgery, the WBC and CRP levels in the ERAS group decreased rapidly, indicating that preoperative and intraoperative ERAS measures could help the child recover after removing the lesion. There is not much difference in IL-6 between the two groups, which may be due to the use of laparoscopic minimally invasive technology in both groups, which effectively controls postoperative stress levels, or the set time points for blood sampling are too few to accurately detect the changes in postoperative inflammatory indicators. The ERAS protocol plays an important role in helping patients recover and alleviate postoperative stress (9). Anna W (12) found that during surgical stress response, the mRNA levels of inflammatory pathway genes (such as IL6, TNF, and nuclear factors) change. CRP is an acute phase protein synthesized by liver cells, discovered by Tillett and Francis in 1930 (13). CRP reflects the impact of trauma on the body and is related to the severity of tissue damage. After surgery, CRP concentration usually increases rapidly within 4–12 h and reaches its peak within 24–72 h, lasting up to 2 weeks. The increase of proinflammatory cytokine IL-6 after surgery is related to insulin resistance (14), and its sensitivity is higher than CRP. Jawa RS (2011) pointed out that the increase of IL-6 after surgery is related to the degree of tissue damage (15), including the surgical approach, laparoscopy and open surgery, the complexity of surgery, cholecystectomy, and colectomy. (2) On the third day after surgery, the IL-6 levels in the ERAS group were significantly lower than those in the TS group, indicating that ERAS related regimens have certain effects in reducing stress and accelerating recovery in children. Preoperative oral administration of carbohydrates improves insulin resistance and reduces hunger, which may be beneficial for the patient's inflammatory response, immune function, and postoperative recovery, without increasing the risk of anesthesia and aspiration during the surgery (16). In addition, preoperative induction anesthesia and medication before anesthesia can calm the child, reduce anxiety, optimize intraoperative hemodynamic stability, and reduce postoperative side effects. The chief surgeon communicates fully with the anesthesiologist, which helps the anesthesiologist determine the dosing time, reduce anesthesia dosage, and accelerate postoperative recovery. Simultaneously, the application of intraoperative insulation devices can reduce the side effects of anesthesia, such as hypothermia, decreasing the impact on coagulation pathways and surgical stress (17). A series of perioperative strategies have played a promoting role in helping children recover postoperative inflammatory indicators.

Accelerate postoperative intestinal recovery, shorten hospital stays, and reduce hospital expenses. Three indicators in the ERAS group are superior to those in the TS group, which are important indicators for accelerating the recovery of pediatric patients and a comprehensive reflection of the ERAS perioperative plan. We analyzed them based on the following aspects: (1) Preoperative: Shorten fasting time. Because children's stomach capacity is smaller than that of adults, traditional preoperative fasting with water can make children hungry and increase anxiety. Drinking 12.5% glucose water 2 h before surgery, and abstaining from water 2 h before surgery, can reduce this anxiety and help reduce blood pressure fluctuations and reduce intravenous fluid replacement in children. Preoperative mechanical bowel preparation can also increase the loss of intestinal fluid, so we should avoid unnecessary bowel preparation; No catheter before an operation and no drainage tube during the operation can reduce the discomfort and pain of children, which is conducive to getting out of bed early (18). In addition, indwelling abdominal drainage tubes may also increase complications such as incision infection and small intestinal obstruction (19). (2) Intraoperative: Control fluid velocity and intake, develop personalized fluid replacement plans. Low blood volume can lead to insufficient organ perfusion, while high blood volume can increase the incidence of postoperative intestinal obstruction (20). Postoperative: Early bedridden activities can distract children's attention, reduce pain, shorten fasting time, reduce fluid replacement, and promote gastrointestinal recovery.

Postoperative analgesia is a crucial measure for children, as non-opioid drugs and local analgesia can effectively reduce gastrointestinal suppression and reduce stress reactions (7). This study achieved good analgesic effects by combining ropivacaine incision analgesia with acetaminophen suppository anal tamponade. Under the premise of effective analgesia, avoiding analgesic drugs that inhibit gastrointestinal peristalsis can accelerate the recovery speed of children. The analgesic effect of the ERAS group at 3 h and 6 h after surgery is significantly better than that of the TS group, which is closely related to ropivacaine incision analgesia and stabilizing the patient's emotions. Local anesthesia can effectively reduce postoperative incision pain in children, alleviate their resistance to emotions, and reduce the use of early postoperative painkillers. We did not find any differences in complications between the two groups of children. Both groups of children suffered from partial completeness intestinal obstruction and abdominal pain after surgery. The analysis of the main causes found that we need to take into account the intra-abdominal conditions of children during surgery while shortening the fasting time according to ERAS. For children with perforated appendicitis and high accumulation of pus between the intestines, fasting with water and a liquid diet should be appropriately extended. The stimulation of pus and inflammatory reactions can slow down the recovery of intestinal peristalsis, and early eating may cause symptoms such as abdominal pain and vomiting. The results of this study may be due to the fact that ERAS accelerates the recovery of children without affecting complications, and may also be related to the small sample size of this study. More clinical evidence is needed to determine the impact of ERAS on the incidence of complications and readmission rates. This study found that the satisfaction of parents in the ERAS group was higher than that in the TS group, mainly because ERAS education can fully reduce the emotional impact of parents' anxiety on children, and accelerate the progress of preoperative (chest x-ray, electrocardiogram, etc.) examinations, allowing parents to fully participate in treatment. In addition, the ERAS program greatly solves the problems of preoperative and postoperative hunger and crying, postoperative pain, long infusion time, high dosage, and the impact of crying among patients in the same ward. It increases the interaction and communication between patients and medical staff, reflects more humanistic care, helps promote harmonious progress of doctor-patient relationships, and establishes a mutual trust medical environment.

In summary, the application effect of the ERAS concept in complex appendicitis in children is better than that of traditional perioperative preparation. It can promote postoperative recovery, shorten hospitalization time, and improve comfort and parental satisfaction of children. It has a positive role in reducing patients' economic burden and promoting the good development of doctor-patient relationships. The smooth progress of the accelerated rehabilitation plan requires the joint cooperation of clinical doctors, nursing staff, anesthesiologists, and relevant clinical departments. At the same time, after fully understanding the disease situation and treatment plan, the family members of the patient can actively cooperate with the doctor to participate in clinical treatment, reduce their anxiety, increase communication and trust between doctors and patients, help the patient overcome fear, and better cooperate with treatment.

## Conclusion

In conclusion, ERAS has obvious advantages in patients with appendicitis after surgery over traditional surgery in reducing inflammation-related indicators levels, promoting postoperative recovery, analgesia, shortening the first exhaust time, shortening the length of hospital stay, saving hospitalization costs and improving parental satisfaction, and does not increase the incidence of postoperative complications. This study demonstrates that ERAS appears to be a safe and feasible postoperative recovery option for patients with complex appendicitis.

## Data Availability

The raw data supporting the conclusions of this article will be made available by the authors, without undue reservation.
